# Complement dysregulation associated with a genetic variant in factor H-related protein 5 in atypical hemolytic uremic syndrome

**DOI:** 10.1007/s00467-023-06184-6

**Published:** 2023-11-13

**Authors:** Sigridur Sunna Aradottir, Ann-Charlotte Kristoffersson, Erik Linnér, Diana Karpman

**Affiliations:** Department of Pediatrics, Clinical Sciences Lund, Lund University, 22185 Lund, Sweden

**Keywords:** Atypical hemolytic uremic syndrome, Complement, Factor H-related protein 5, Hemolysis, Sequencing

## Abstract

**Background:**

Atypical hemolytic uremic syndrome (aHUS) can be associated with mutations, deletions, or hybrid genes in factor H-related (FHR) proteins.

**Methods:**

A child with aHUS was investigated. Genetics was assessed by Sanger and next generation sequencing. Serum FHR5 was evaluated by immunoblotting, ELISA, and by induction of rabbit red blood cell hemolysis in the presence/absence of recombinant human rFHR5. Mutagenesis was performed in HEK cells.

**Results:**

A heterozygous genetic variant in factor H-related protein 5 (*CFHR5*), M514R, was found in the child, who also had a homozygous deletion of *CFHR3/CFHR1*, and antibodies to factor H, as well as low levels of C3. Patient serum exhibited low levels of FHR5. In the presence of rabbit red blood cells, patient serum induced hemolysis which decreased when rFHR5 was added at physiological concentrations. Similar results were obtained using serum from the father, bearing the *CFHR5* variant without factor H antibodies. Patient FHR5 formed normal dimers. The *CFHR5* M514R variant was expressed in HEK cells and minimal secretion was detected whereas the protein level was elevated in cell lysates.

**Conclusions:**

Decreased secretion of the product of the mutant allele could explain the low FHR5 levels in patient serum. Reduced hemolysis when rFHR5 was added to serum suggests a regulatory role regarding complement activation on red blood cells. As such, low levels of FHR5, as demonstrated in the patient, may contribute to complement activation.

**Graphical abstract:**

**Figure:**
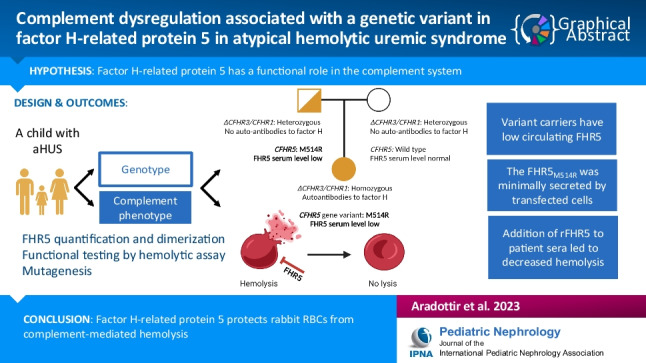
A higher resolution version of the Graphical abstract is available as Supplementary information

**Supplementary information:**

The online version contains supplementary material available at 10.1007/s00467-023-06184-6.

## Introduction

Atypical hemolytic uremic syndrome (aHUS) is a complement-mediated kidney disease characterized by the development of non-immune hemolytic anemia, thrombocytopenia, and kidney injury [1]. The disease is rare and may have a recurring and chronic nature. aHUS can be associated with heterozygous genetic variants in genes encoding complement factors or regulators [2]. These include gain-of-function variants in C3 [3] or factor B [4] which can result in the formation of a stable C3 convertase resistant to factor H-mediated dissociation, or loss-of-function variants in complement regulators including factor H [5], membrane-cofactor-protein/CD46 [6] and factor I [7], allowing excess complement activation via the alternative pathway.

Mutations in factor H have been localized to the C terminal of the protein. The C terminal has properties associated with host cell recognition thereby directing complement to be activated on foreign surfaces but not on host cells [8]. Genetic variants in this region of the protein can disturb the differentiation between self and non-self and thereby allow complement to become activated on host cells. Similarly, some patients with aHUS have been shown to have antibodies to factor H, mostly directed to the C terminal [9], and these are associated with deletions, genomic rearrangements, and hybrid genes involving factor H-related (FHR) proteins [10, 11]. Homozygous deletions of *CFHR3* and *CFHR1* have been described in patients with antibodies to factor H. Whereas factor H consists of 20 short consensus repeats (SCRs), with the surface recognition C terminal domain at SCRs 19-20, factor H-related proteins consist of 4-9 SCRs with a certain degree of homology to factor H, and with dimerization motifs [12, 13].

FHR5 is a 65 kDa protein consisting of nine SCRs and capable of binding C3 and heparin. Its exact function in the complement pathway is still a matter of investigation. Studies using higher than physiological concentrations have shown it to regulate complement activation [14], while other studies have demonstrated a role in promoting complement activation by competing with factor H [15] and by contributing to formation of the C3 convertase [16]. Genetic variants in *CFHR5* have been mostly associated with C3 glomerulopathy and one subtype of the disease is termed CFHR5-nephropathy [17]. However, genetic variants have also been identified in aHUS [18–20].

In this study, a rare *CFHR5* variant in aHUS, M514R, was investigated. This variant was previously reported in C3 glomerulopathy [21] and age-related macular degeneration [22], but its phenotype has not been described. The aim of the study was to investigate the *CFHR5* M514R variant regarding complement activation or regulation on red blood cells.

## Methods

### Case study

A five-year-old girl presented in 2012 with hemolytic anemia (hemoglobin 58, reference value 100–150 g/L; lactate dehydrogenase 25, reference value 2.2–5.3 μkat/L; bilirubin 31, reference value 5–25 μmol/L, direct antiglobulin test negative), thrombocytopenia (platelet count 22, reference value 125–340 × 10^9^/L), and kidney injury (creatinine 124, reference value 14–42 μmol/L) without diarrhea. A fecal sample for enterohemorrhagic *Escherichia coli* was negative. Initial assessment of complement values showed C3 0.63, reference value 0.77–1.38 g/L and C3d 14, reference value < 5 mg/L. Qualitative assays of complement function via the classical and alternative pathways, assessed in gels using sheep and guinea pig red blood cells (RBCs), respectively, were repeatedly normal. ADAMTS13 was normal. These assays were performed at Skåne University Hospital laboratories.

At the initial presentation, antibodies to factor H were detected in the child’s serum. Initial anti-factor H antibody levels were 16,460 E/mL. The levels did not correspond to international units as a standard assay had not yet been developed [9]. The presumptive diagnosis was aHUS and the child was initially treated with daily fresh frozen plasma infusions to which she responded well, followed by mycophenolate mofetil (MMF) and plasma treatment at prolonged intervals up to every other week. The patient was not treated with eculizumab. Eighteen months after the initial episode, the patient had a recurrence during a febrile infection and 1 month later an additional recurrence. These recurrences were treated with plasma exchanges, altogether 9 exchanges three times a week. During this period an additional mild recurrence occurred. Treatment with cyclophosphamide pulses every third week was given during a 5-month period followed by MMF. The treatment protocol was in accordance with a multicenter study of aHUS patients with antibodies to factor H [23]. In 2014, the Clinical Immunology Laboratory in Lund in collaboration with other European laboratories developed a standardized assay and the patient’s anti-factor H value was 2540 E/mL. Levels decreased continuously over the years, in 2015: 840, 2019: 710, 2022: 570, and in 2023: 260 E/mL. The detection limit is 99 E/mL. Likewise, C3 and C3d levels normalized after therapeutic plasma exchanges and remained normal thereafter.

The girl did not experience any recurrences after this and MMF was slowly tapered and discontinued when she was 15 years old. Kidney function was assessed by glomerular filtration rate measured with iohexol clearance was 97 mL/min/1.73 m^2^ at 9 years and 79 mL/min/1.73 m^2^ at 16 years. The patient does not have proteinuria or hypertension.

The child’s mother had, during childhood, an unclear transient episode of thrombocytopenia. The child’s father has not had any symptoms associated with HUS. There was no known family history of atypical HUS.

### Controls

Apparently healthy adults (*n* = 9) were controls, and four of these were female.

The study of the patient, her parents, and healthy controls was performed with the approval of the Swedish Ethical Review Authority, approval no. 2021-04438. Informed written consent was obtained from the patient, her parents, and the healthy controls.

### Blood samples

Whole blood in EDTA tubes was used for DNA extraction as previously described. Serum samples were centrifuged after 1-h incubation at room temperature, aliquoted, and stored at − 80 °C until used. Samples from the patient were collected during remission.

### Genetic analysis and mutation screening

DNA samples were subject to Sanger sequencing (Eurofins Genomics, Konstanz, Germany) and analyzed using BioEdit (Ibis Biosciences, Carlsbad, CA). In addition, whole exome sequencing and whole genome sequencing were carried out at the Center for Molecular Diagnostics, Skåne University Hospital as previously described [24]. The gene panel included *CFH*, *CFI*, *CD46, C3*, *C5*, *CFB, CFP*, *CLU, CFHR1-5*, *ADAMTS13*, *THBD*, *DGKE*, and *PLG*. Allele frequencies in the normal population were obtained from the Genome Aggregation Database (gnomAD, http://gnomad.broadinstitute.org/).

### Determination of FHR5 levels

FHR5 concentration was assayed by an ELISA developed in-house. A MaxiSorp® plate (Thermo Fisher Scientific, Roskilde, Denmark) was coated overnight at 4 °C with a polyclonal goat anti-hFHR5 (R&D Systems, Minneapolis, MN) for capture. The antibody was diluted in 0.1 M carbonate buffer (pH 9.6) to a final concentration of 1 µg/mL. The wells were blocked for 1 h with bovine serum albumin (Sigma, St. Louis, MO). Recombinant hFHR5 was used as the standard (catalog number: 3845-F5, R&D Systems). The subsequent 1-h incubations were performed at room temperature and washed with PBS-Tween (Medicago, Uppsala, Sweden). Monoclonal mouse anti-hFHR5-antibody (1 µg/mL, R&D Systems) was followed by goat anti-mouse IgG: HRP (1:1000 Dako, Glostrup, Denmark) and tetramethylbenzidine (TMB) (Dako, Carpinteria, CA). The reaction was terminated with sulfuric acid (H_2_SO_4_, Sigma-Aldrich) and absorbance measured at 450 nm using GloMax Discover (Promega, Madison, WI).

### Determination of FHR5 size

FHR5 size was determined by immunoblotting under non-reducing conditions. Proteins were separated by sodium dodecyl sulfate-polyacrylamide gel electrophoresis (SDS-PAGE, 10%, Mini-PROTEAN®, Bio-Rad Laboratories, Hercules, CA) and transferred by electroblotting to a polyvinylidene difluoride membrane (PVDF, Bio-Rad). Recombinant hFHR5 was used as the control. Polyclonal goat anti-hFHR5-antibody diluted 1:2000 was used for detection, followed by rabbit anti-goat IgG: HRP antibody (Dako) at 1:2000. Detection was performed using chemiluminescence (Pierce ECL2, Rockford, IL) and imaged using ChemiDoc™ Touch, Bio-Rad.

### Hemolysis of rabbit red blood cells

Rabbit red blood cells are highly susceptible to complement-mediated hemolysis as they have decreased sialic acid on their membrane and thus do not bind the complement regulator factor H [25]. Rabbit RBCs (5 × 10^8^/mL, Håtunalab) in gelatin veronal buffer (GVB) with Mg-ethylene glycol tetraacetic acid (EGTA) 0.1 M were incubated with serum diluted 1:10. The total volume of the samples was 100 µl including 20 µl rabbit RBCs, 70 µl Mg-EGTA buffer with or without added recombinant hFHR5 and 10 µl serum (hFHR5 added in the final step or first combined with serum, preincubated for 10 min, and then added to the tube). Added hFHR5 was 50 or 500 ng in the total volume of 100 µl, in which serum was diluted 1:10; as such the lower amount corresponds to physiological serum concentrations of 3–6 µg/mL.

Samples were incubated for 1 h on a shaker at 37 °C, with additional manual shaking every 10 min. GVB-EDTA was added (100 µl, Complement Technology, Tyler, Texas) followed by a centrifugation step. The supernatant was collected, and absorbance was measured at 405 nm using GloMax Discover.

### Analysis of CFHR5 dimerization

Serum samples were diluted 1:5 in PBS (GE Life Sciences, Uppsala, Sweden) and added to centrifugal tubes (Amicon® Ultra-4 Centrifugal Filter Unit, Sigma-Aldrich) with 100 kDa cutoff. Samples were washed with PBS twice. The concentrate as well as the filtrate were analyzed by SDS-PAGE and immunoblotting. Recombinant hFHR5 was used as the control.

### Mutagenesis

The pcDNA3.1(-)Myc/His A plasmid (ampicillin-resistant, Invitrogen, Life Technologies, Waltham, MA) containing wild-type *CFHR5* gene was kindly provided by M. Pickering (Imperial College London, Faculty of Medicine). A vector sequence containing the mutation was separately obtained (Eurofins Medigenomix). The plasmid containing the wild-type sequence and the vector containing the mutated sequence were cleaved at two sites by AflII (New England Biolabs, Ipswich, MA). The resulting cleaved DNA fragment was 822 bp. The fragments and inserts were separated on an agarose gel and purified using QIAquick Gel Extraction Kit (QIAGEN, Hilden, Germany). Dephosphorylation of the vector solution was performed with the rAPid Alkaline Phosphatase kit (Roche Diagnostics, Mannheim, Germany) to prevent auto-ligation. The two fragments (mutant insert and vector without the wild-type sequence) were ligated using a T4 DNA Ligase kit (Roche Diagnostics, Indianapolis, IN).

The transformation was performed in *E. coli* (XL-Gold Ultracompetent cells, Agilent Technologies, Santa Clara, CA) and the plasmid was purified (QIAprep® Spin Miniprep Kit, QIAGEN). The correct insertion and the sequence were verified by Sanger sequencing performed at Eurofins Genomics and interpreted.

### Cell transfection

HEK 293 T cells (human embryonic kidney cells, ATCC, Teddington, Middlesex, UK) were seeded and grown in DMEM/high glucose Hyclone medium (GE Healthcare Life Sciences, South Logan, UT), supplemented with 100 U/mL penicillin, 100 µg/mL streptomycin, and 10% fetal bovine serum to approximately 95% confluence before transfection. Transient transfection was performed with Lipofectamine (Invitrogen, Life Technologies, Waltham, MA) according to the manufacturer’s instructions. A control well without added DNA was included. Twenty-four hours after transfection, the medium was changed to Optimem (Thermo Fisher Scientific), and cells were cultured for an additional 72 h. The media were collected, supplemented with protease inhibitors cOmplete Mini without EDTA (Roche Diagnostic, Mannheim, Germany) and centrifuged to separate supernatant and cell debris. The supernatant was concentrated 25 × to 200 µL using a 10 kDa filter (Ultra-4 Centrifugal Filter Units, Merck Millipore, Billerica, MA). The cells were diluted in RIPA buffer for protein extraction (Sigma) with cOmplete Mini without EDTA, scraped from the wells, and the dilution underwent three freeze-thaw cycles to enhance lysis.

### Statistics

Non-parametric Wilcoxon signed-rank test was used to compare paired sera from the same individual with and without added FHR5. Statistical analysis was performed using GraphPad Prism 8 software (version 8.4.3, GraphPad Software, La Jolla, CA).

## Results

### Patient workup

Genetic workup showed that the patient was homozygous for a deletion of *CFHR3/CFHR1* and heterozygous for a rare variant in *CFHR5* c.1541T > G; p.M514R. The father was shown to be a heterozygous carrier of the *CFHR5* variant M514R and for the deletion of *CFHR3/CFHR1*. The mother did not carry the *CFHR5* M514R variant but was heterozygous for the *CFHR3/CFHR1* deletion. Neither one of the parents had antibodies to factor H.

**Fig. 1:**
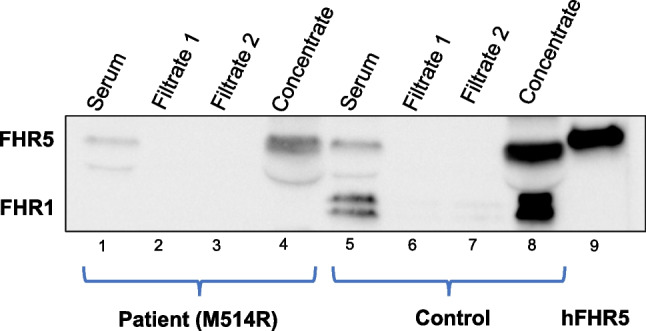
Immunoblot of factor H-related protein 5 in serum. Lanes 1–4 show the patient’s serum. Lane 1: Patient serum exhibiting a weak band corresponding to factor H-related protein 5 (FHR5) but no bands corresponding to FHR1. Lanes 2 and 3: Serum filtrate < 100 kDa lacking bands indicating that all circulating FHR5 was dimerized. Lane 4: Serum concentrate > 100 kDa, after electrophoresis separation to 65 kDa subunits, indicating that FHR5 was initially dimerized (at 130 kDa). Lanes 5–8 show control serum. Lane 5: Control serum showing bands corresponding to FHR5 and FHR1. Lanes 6 and 7: Filtrate of control serum lacking bands indicating that all circulating FHR5 was dimerized. Lane 8: Serum concentrate > 100 kDa, after electrophoresis separation to 65 kDa subunits, indicating that FHR5 was dimerized. Lane 9: Recombinant human FHR5. The band is somewhat higher than serum FHR5 presumably due to differences in glycosylation

FHR5 levels in the patient’s and the father’s serum measured by ELISA were low, in the patient: 0.76 and 0.8 µg/mL (two separate samples), in the father: 0.8 µg/mL, compared to controls (*n* = 5) ranging between 1.49 and 3.1 µg/mL. Although the size was normal, only a very weak band was detected by immunoblotting, corresponding to FHR5. The antibody to FHR5 also detects FHR1 but no bands corresponding to this protein were detected in the patient’s serum (Fig. 1).

An experiment was designed to determine if FHR5 in patient serum could form dimers. Using a cutoff filter of 100 kDa FHR5 dimers (approximately 130 kDa) would be collected in the concentrate and be separated by SDS-PAGE to the 65 kDa subunits, as shown in Fig. 1. FHR5 in patient serum formed dimers.

### FHR5 decreases hemolysis of rabbit RBCs

**Fig. 2:**
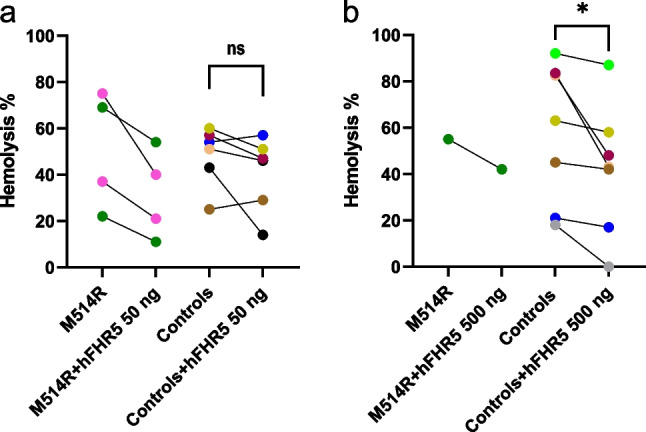
Hemolysis induced by patient, father’s and normal serum in the presence of factor H-related protein 5.** a** Serum from the patient (pink) and father (green), both bearing the *CFHR5* variant M514R, induced hemolysis of rabbit red blood cells, inhibited by the addition of physiological concentrations of recombinant human factor H related 5 (hFHR5). Control sera (*n* = 6) did not exhibit a significant change in hemolysis when hFHR5 was added. ns, not significant.** b** Serum from the father bearing the *CFHR5* variant M514R induced hemolysis that was reduced when hFHR5 was added at higher than physiological concentrations. Similar results were obtained using control sera (*n* = 7). *P < 0.01

Combining rabbit red blood cells (RBCs) with patient serum induced hemolysis which decreased when recombinant hFHR5 was added at physiological concentrations (Fig. 2a). Similar results were obtained using serum from the father, bearing the *CFHR5* M514R variant, without antibodies to factor H. The father’s serum was also combined with hFHR5 at higher concentrations, also showing a decrease in hemolysis (Fig. 2b). Using control sera, the degree of hemolysis decreased when adding high concentrations of hFHR5 but did not change significantly when adding physiological concentrations (Fig. 2). Adding hFHR5 to the cells before adding the serum, or combined with serum, gave similar results.

### Studies of the transfected CFHR5 M514R variant

**Fig. 3:**
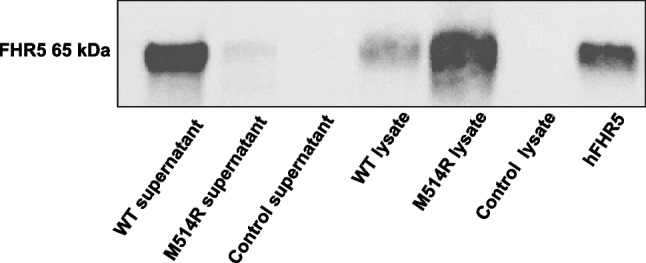
Factor H-related protein 5 wild-type and mutant proteins in cell supernatants and lysates. Factor H-related protein 5 was assayed by immunoblotting in transfected cell supernatants and lysates containing the wild-type sequence and the mutant M514R variant. Wild-type supernatant exhibited a strong band corresponding to FHR5. The supernatant from cells transfected with the CFHR5 M514R variant exhibited a very weak band. Control supernatants from HEK cells that were not transfected did not exhibit a band corresponding to FHR5. The FHR5 band was stronger in the lysates of the cells containing the M514R mutant variant compared to the wild-type, as the mutant variant was minimally secreted. hFHR5, recombinant human factor H-related protein 5; WT, wild-type; M514R, transfected mutant variant

In order to characterize the phenotype of the mutated protein, the *CFHR5* M514R variant was expressed in HEK cells. Levels of the wild-type FHR5 protein in the cell supernatants were assayed by ELISA and detected at a median of 4176 ng/mL (three separate transfections), whereas the mutant variant was secreted at 0.21-fold of the wild-type (median of two separate transfections). Cell lysates from the transfected cells containing the mutant variant showed 8.5-fold higher levels than the wild-type (median of the wild-type in lysates 118 ng/mL). These results were confirmed by immunoblotting showing a strong FHR5 band in the supernatant from HEK cells expressing the wild-type protein, but a very weak band in cells expressing the mutant variant M514R (Fig. 3). In contrast, the cell lysates from HEK cells expressing the wild-type protein showed a weak band and the lysates from cells expressing the mutant variant showed a stronger FHR5 band. Taken together, these results indicate that the mutant variant accumulated intracellularly. The non-transfected control supernatants or cell lysates did not contain FHR5 protein (Fig. 3).

## Discussion

Genetic variants in *CFHR5* have been detected in patients with complement-mediated kidney disease such as aHUS [19, 20], C3 glomerulopathy, and immune complex-associated membranoproliferative glomerulonephritis [17, 26]. The detection of a rare variant does not, however, indicate its involvement in the disease process. Here, we describe the phenotype of the *CFHR5* variant M514R showing that it is minimally secreted from cells. Negligible amounts of the product of the mutant allele would therefore be present in the circulation. FHR5 has been suggested to be either an activator or a regulator of complement [27]. The patient described herein had a homozygous deletion of *CFHR3/CFHR1* and initially high levels of antibodies to factor H. The latter could explain her predisposition to develop aHUS. Low levels of FHR5 were demonstrated in the patient’s serum, most probably representing translation of the normal allele, which could form FHR5 dimers. The addition of physiological concentrations of recombinant human FHR5 to patient serum decreased hemolysis of rabbit RBCs. This was also demonstrated using the father’s serum (carrying the same *CFHR5* variant without antibodies to factor H). The results suggest FHR5 has a regulatory role on the surface of RBCs and its deficiency could thereby contribute to complement activation leading to hemolysis.

Two factors in the patient’s serum may have played an important role in the development of aHUS; the antibodies to factor H and the *CFHR5* variant described herein. The antibodies to factor H most likely contributed to the development of disease as she responded well to plasma exchange and immunosuppressive treatment. However, we speculate that the decreased FHR5 could also promote complement activation as FHR5 was shown here to protect RBCs from complement-mediated lysis. Interestingly, the same variant was previously described in a patient with C3 glomerulopathy and circulating C3 nephritic factor [21]. The presence of a variant in genes encoding complement factors or regulators may increase the risk of disease but does not solely determine the phenotype of disease. Our group has previously shown that monozygotic twins with a gain-of-function variant in *CFB*, encoding complement factor B, differed regarding the development of aHUS [24]. Moreover, the same genetic variant may be associated with aHUS in one individual and C3 glomerulopathy in another [28]. In addition to genetic composition, other factors may play a role, such as environmental exposures (i.e., vaccinations and infections.) as well as epigenetic factors.

FHR5 colocalizes with glomerular C3 deposits in the kidney [29] and binds to C3b in vitro [30]. These properties suggest a role in interacting with C3. A previous study showed that FHR5 could inhibit complement activation by functioning as a cofactor for factor I-induced C3b degradation to iC3b and by inhibiting C3 convertase activity, both assays performed in the fluid phase and with higher than physiological FHR5 concentrations [14]. For this reason, the relevance could be questioned. In the current study, we show that FHR5 has a complement regulatory role on RBCs using physiological concentrations and sera with the M514R variant. Similarly, a study of patients with CFHR5 nephropathy with duplication of exons 2 and 3 in *CFHR5* demonstrated that the mutant variant exhibited decreased binding to chicken RBCs [17]. If FHR5 is a complement regulator on RBCs then its deficiency due to lack of cellular secretion (as presented herein) or decreased binding (as shown in [17]) could allow excess complement-mediated hemolysis to proceed.

In contrast, other studies have suggested that FHR5 could be a complement activator showing that it interferes with factor H binding to C3b and that this interaction is influenced by the presence of surface glycosaminoglycans [15]. The discrepancies may be related to the assay used and the presence of other serum proteins. There is also the possibility that FHR5 has dual roles, both an activating and a regulatory role in the complement system depending on the microenvironment and its activity in the fluid phase versus the cell surface.

The mutant variant of *CFHR5*, M514R, studied here, led to low circulating serum levels. The addition of recombinant human FHR5 seemed to have a protective role on rabbit RBCs, when higher than physiological concentrations were used, or at physiological concentrations in the patient’s serum. Thus, low serum levels could contribute to complement-mediated hemolysis. We do not suggest, however, that the *CFHR5* variant plays a major role in the disease phenotype in this patient. It seems that levels of anti-factor H antibodies play a more important role in the induction of disease. However, the *CFHR5* variant may have an additional role in promoting complement activation once the disease has been triggered.

### Supplementary information

Graphical abstract (PPTX 976 KB)

## Data Availability

All data are available within the manuscript and its supplement. Genetic data were also deposited in Zenodo (https://doi.org/10.5281/zenodo.8124309).
