# Supplementary material for: Complement dysregulation associated with a genetic variant in factor H-related protein 5 in atypical hemolytic uremic syndrome
Source: Pediatr Nephrol. 2023 Nov 13;39(4):1105–11. doi: 10.1007/s00467-023-06184-6 (PMC10899364; doi:10.1007/s00467-023-06184-6)
Supplement: Supplementary file 1 — Graphical abstract (PPTX 976 KB) [file 467_2023_6184_MOESM1_ESM.pptx]

## Slide 1
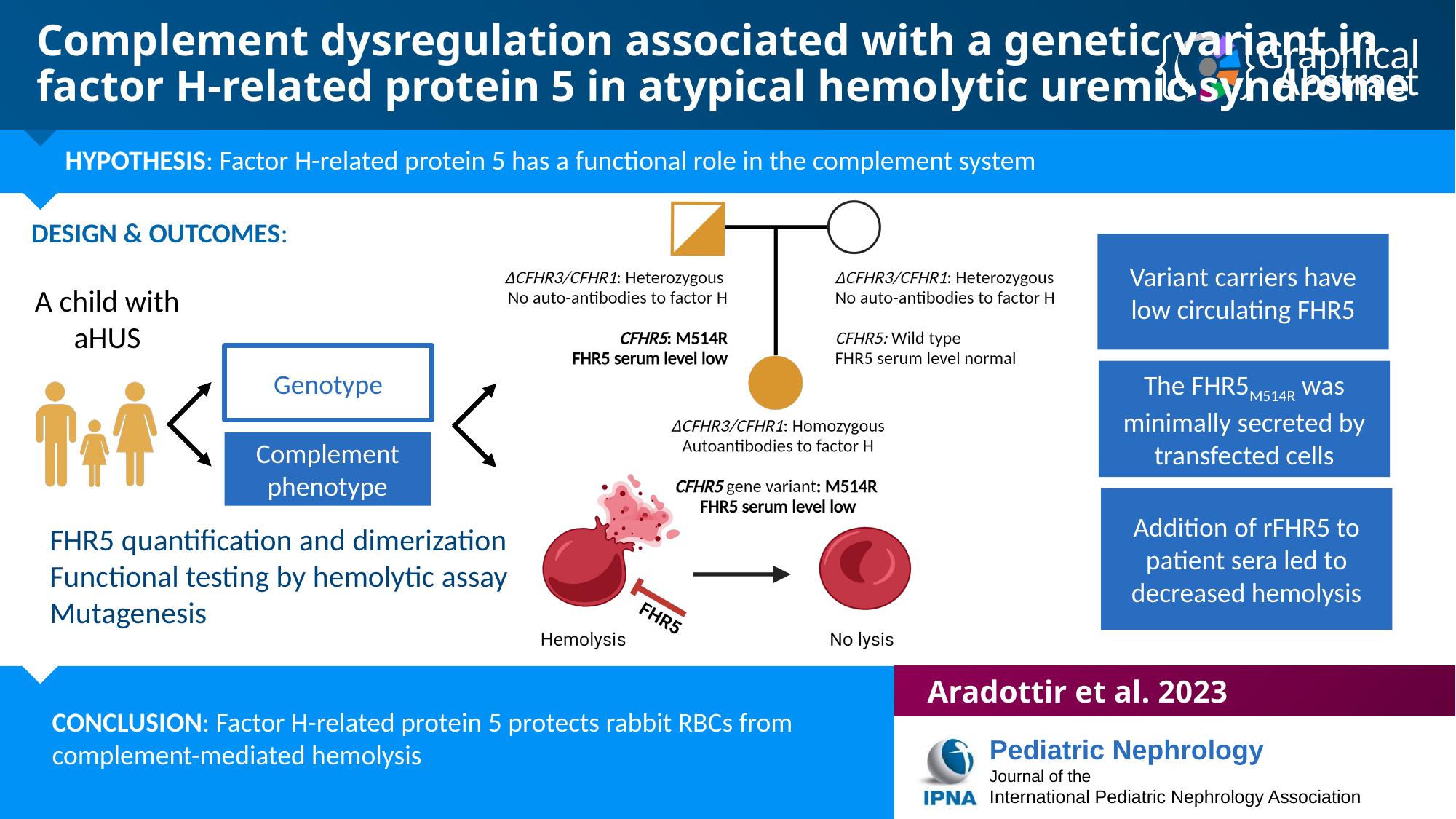

Complement dysregulation associated with a genetic variant in
factor H-related protein 5 in atypical hemolytic uremic syndrome
HYPOTHESIS: Factor H-related protein 5 has a functional role in the complement system
DESIGN & OUTCOMES:
Variant carriers have low circulating FHR5
A child with aHUS
Genotype
The FHR5M514R was minimally secreted by transfected cells
Complement phenotype
Addition of rFHR5 to patient sera led to decreased hemolysis
FHR5 quantification and dimerization
Functional testing by hemolytic assay
Mutagenesis
Aradottir et al. 2023
CONCLUSION: Factor H-related protein 5 protects rabbit RBCs from complement-mediated hemolysis
